# A family AA5_2 carbohydrate oxidase from *Penicillium rubens* displays functional overlap across the AA5 family

**DOI:** 10.1371/journal.pone.0216546

**Published:** 2019-05-15

**Authors:** Filip Mollerup, Ville Aumala, Kirsti Parikka, Yann Mathieu, Harry Brumer, Maija Tenkanen, Emma Master

**Affiliations:** 1 Department of Bioproducts and Biosystems, Aalto University, Aalto, Finland; 2 Department of Food and Environmental Sciences, University of Helsinki, Helsinki, Finland; 3 Michael Smith Laboratories, University of British Columbia, Vancouver, British Columbia, Canada; 4 Department of Chemical Engineering and Applied Chemistry, University of Toronto, Toronto, Ontario, Canada; Institut National de la Recherche Agronomique, FRANCE

## Abstract

Copper radical alcohol oxidases belonging to auxiliary activity family 5, subfamily 2 (AA5_2) catalyze the oxidation of galactose and galactosides, as well as aliphatic alcohols. Despite their broad applied potential, so far very few AA5_2 members have been biochemically characterized. We report the recombinant production and biochemical characterization of an AA5_2 oxidase from *Penicillium rubens Wisconsin 54–1255* (*Pru*AA5_2A), which groups within an unmapped clade phylogenetically distant from those comprising AA5_2 members characterized to date. *Pru*AA5_2 preferentially oxidized raffinose over galactose; however, its catalytic efficiency was 6.5 times higher on glycolaldehyde dimer compared to raffinose. Deep sequence analysis of characterized AA5_2 members highlighted amino acid pairs correlated to substrate range and conserved within the family. Moreover, *Pru*AA5_2 activity spans substrate preferences previously reported for AA5 subfamily 1 and 2 members, identifying possible functional overlap across the AA5 family.

## Introduction

Biocatalysts developed to date to bolster the utilization of plant biomass have focused on the deconstruction of lignocellulose to sugars that can then be converted to fuels and chemicals [1,2]. On the other hand, the functional derivatization of plant material to make high-value bioproducts is a new area of biomass utilization research. Auxiliary Activity family 5 (AA5) members are copper radical oxidases (CROs) which are attractive targets for this purpose because of their ability to perform oxidation in a chemo-selective manner using only an inexpensive copper ion cofactor and oxygen. The AA5 family includes two subfamilies, namely AA5_1 and AA5_2, comprising characterized glyoxal oxidase and alcohol/carbohydrate oxidase enzymes respectively. So far, only few members of the AA5_2 subfamily (E.C.: 1.1.3.9) have been characterized, including the archetypal galactose oxidase from *Fusarium graminearum* (*Fgr*GaOx). Such galactose oxidases comprise an N-terminal carbohydrate-binding module (CBM32, PF00754), a central catalytic domain containing three of the four copper-ligands (pfam Kelch_1 domain, PF01344), and a C-terminal domain (pfam DUF_1929 domain, PF09118) that provides the fourth copper ligand [3]. They catalyze the two-electron oxidation of C6-OH of D-galactose, generating the corresponding aldehyde while reducing molecular oxygen to hydrogen peroxide [4,5]. The aldehyde product can also be further oxidized to the carboxylic acid through oxidation of the geminal diol derivative of the aldehyde product [6]. While the k_cat_ of *Fgr*GaOx is approximately 100 times higher on D-galactose than galactose-containg polysaccharides, *Fgr*GaOx shows nearly two times higher catalytic efficiency (*k*_cat_/*K*_m_) on galactoglucomannan and galactoxyloglucan compared to galactose [7]. The performance of *Fgr*GaOx on galactose-containg polysaccharides has prompted its use in a broad range of applications [8], including hydrogels and aerogels [9–12], as well as cellulose coatings [13–15].

Previous work in our groups unveiled catalytic diversity within the AA5_2 subfamily beyond the galactose oxidases from *Fusarium sp*. Specifically, two AA5_2 homologs from the phytopathogenic fungi *Colletotrichum graminicola* (*Cgr*AlcOx), and *C*. *gloeosporioides* (*Cgl*AlcOx) were characterized as general alcohol oxidases based on their high enzymatic activity towards aromatic and aliphatic alcohols, rather than carbohydrates [16]. Later, the raffinose oxidase from *C*. *graminicola* (*Cgr*RaOx), containing a PAN_1 domain (PF00024) instead of the N-terminal CBM32 (PF0754) of *Fgr*GaOx, was also reported [6]. In the present study we further investigated the protein sequence space within the AA5_2 subfamily using the catalytic modules from the CAZy database and sequence-function correlations of characterized AA5_2 members. Our analyses led to the selection of *Pru*AA5_2A from *Penicillium rubens Wisconsin 54–1255* (strain ATCC28089, UniprotKB: B6HHT0), which displayed dual activity preference on glycolaldehyde dimer and galactose-containing oligosaccharides, consistent with diverse biological functions.

## Materials and methods

### Chemicals and enzymes

Wild-type galactose oxidase from *Fusarium graminearum* was produced in *Pichia pastoris* KM71H and purified as previously described [17]. Horseradish peroxidase (P8375) and catalase from bovine liver (C40) were purchased from Sigma. If not otherwise specified, all chemicals and carbohydrate substrates were purchased from Sigma-Aldrich (USA). Galactoxyloglucan from tamarind was purchased from Megazyme (Ireland).

### Sequence analyses

Fifty-two amino-acid sequences of fungal AA5_2 members and five amino-acid sequences of fungal AA5_1, corresponding to characterized members in the literature, were extracted from the public version of the CAZy database (http://www.cazy.org/AA5.html) [18]. In addition, homologs of CAP96757 were retrieved from the JGI Mycocosm portal [19] by blasting its full length sequence onto the Ascomycota. A total of three sequences with a percentage of identity superior to 60% were included in the analysis. Where present, signal peptides and additional modules, such as carbohydrate-binding modules, were removed to isolate the catalytic modules for subsequent analyses. A multiple sequence alignment was performed using MUSCLE [20] and a maximum likelihood phylogenetic tree was produced using RAxML v.8, with a 100 bootstrap, located on The CIPRES Science Gateway portal [21] (www.phylo.org). Subfamilies were inferred based on their bootstrap values (>75) and the tree was formatted using Figtree.

To identify amino acid positions likely to contribute to substrate range, an alignment was also performed for functionally characterized AA5_2 members, including the alcohol oxidase (AlcOx) (GenBank: EFQ30446.1) and raffinose oxidase (RaOx) (GenBank: EFQ36699) from *Colletotrichum graminicola M1*.*001* (Table 1). In addition, Phyre2 was used to generate structure predictions for *Pru*AA5_2A and *Cgr*RaOx based on homology-modeling [22]. Amino acid differences were mapped to the models and the crystal structures of *Fgr*GaOx (PDBID 1GOG) [3] and *Cgr*AlcOx (PDBID 5C92) [16]. They were then grouped according to function, where Group 1 included catalytic residues and copper-ligands, Group 2 include amino acid positions implicated in substrate range, and Group 3 included amino acid positions identified through mutagenesis to increase catalytic activity or stability. All figures were prepared with UCSF Chimera.

**Table 1:** Amino acids and positions within characterized AA5_2 sequences that are implicated in catalysis and substrate preference.

*F*. *graminearum*					Corresponding amino acid in		
Catagory	Position	Amino acid	Reported function	Reference(s)	*Cgr*RaOx	*Cgr*AlcOx	*Pru*AA5_2
**Catalysis**	194	F	π-π interaction with F227 and W290	[39]	F	W	Y
	228	C	C228-Y272 redox cofactor	[3,5,40]	C	C	C
	272	Y	C228-Y272 redox cofactor, copper ligand	[3,5,40]	Y	Y	Y
	441	F	π-π stacking interaction with Y495	[3]	F	F	F
	464	F	π-π stacking interaction with Y495	[39]	F	F	F
	495	Y	Catalytic tyrosine, copper ligand	[3,5]	Y	Y	Y
	496	H	Copper ligand	[3,5]	H	H	H
	581	H	Copper ligand	[3,5]	H	H	H
**Substrate preference**	290	W	π-π stacking interaction with Y272 and F194, hydrogen bonding with D-galactose	[3,5,16,34,40–42]	Y	F	W
	326	Q^a^	Hydrogen bonding with R330	[34,41]	A	G	D
	329	Y	Hydrogen bonding with D-galactose and R330	[34]	W	M	Y
	330	R	Bidentate hydrogen bonds with D-galactose	[34,37,41]	R	F	R
	405	Y	Hydrogen bonding with Y495	[37]	Y	Y	Y
	406	Q^a^	Hydrogen bonding with D-galactose	[34,37]	S	T	E
**Catalytic efficiency**	383	C^a^	C383S increases V_*max*_ on D-galactose by 1.75x and lowers *K*_*M*_ by 3.6x	[35,36]	C	C	S
	436	Y^a^	Y436H increases V_*max*_ on D-galactose by 2x	[35]	K	Y	A
	494	V^a^	V494A increases V_*max*_ on D-galactose by 1.75x	[35,36,41]	N	N	V

Amino acid positions correspond to the archetypal galactose oxidase from *Fusarium graminearum*.

^a^Amino acids not fully conserved among AA5_2 sequences from other Fusarium species (Fig A in S1 File).

Based on the sequence analyses, *Pru*AA5_2A from *Penicillium rubens* (strain ATCC 28089 / DSM 1075 / NRRL 1951 / Wisconsin 54–1255; Uniprot: B6HHT0; GenBank: 96757.1) was selected for recombinant protein production and characterization.

### Gene synthesis, cloning, expression and purification of *Pru*AA5_2A

Prior to gene synthesis, the native signal sequence of *Pru*AA5_2A was predicted using the SignalIP server [23] and removed from the amino acid sequence. The gene encoding the resulting protein sequence, including prosequence, was optimized for expression in *P*. *pastoris* and then synthesized and cloned into pPICZalpha by Genscript (NJ, USA). Selection of the *P*. *pastoris* transformants and bioreactor expression and purification of *Pru*AA5_2A were performed as previously described [17]. Briefly, the expression vector encoding *Pru*AA5_2A was transformed into *P*. *pastoris* SMD1168H by electroporation [24]. Transformants were then selected on YPD agar plates containing Zeocin (100 μg/ml) and screened for protein expression through colony blotting; supernatant samples from small scale cultivations (5 mL) were also screened for galactose and raffinose oxidase activity as described below. The transformant showing highest expression of *Pru*AA5_2A was then selected for *Pru*AA5_2A production in a bioreactor system following Invitrogen’s Pichia Fermentation Guidelines with minor modifications [17].

To purify *Pru*AA5_2A, the supernatant recovered from the bioreactor cultivation was adjusted to 1.5 M ammonium sulfate (pH 7.5) and then loaded on to a 20 mL sephacryl-phenol (high substitution) column (GE Lifesciences). Fractions containing *Pru*AA5_2A were then pooled and further purified by affinity chromatography using a 5 mL Ni-NTA column equilibrated with 50 mM sodium-phosphate buffer (pH 7.5) containing 20 mM imidazole and 500 mM NaCl. *Pru*AA5_2A was eluted from the Ni-NTA column by gradually increasing the immidazole concentration from 20 to 500 mM. Fractions containing purified *Pru*AA5_2A were then pooled, and the protein was concentrated and exchanged to 50 mM sodium phosphate (pH 7.5) using a Vivaspin 20 ultracentrifugation unit with a 30,000 MWCO cut-off (Satorius, Germany). The purity and molecular mass of *Pru*AA5_2A were assessed by SDS-PAGE using a gel imaging system and Image Lab software from Bio-Rad laboratories (USA). The protein concentration was determined using the Bradford protein assay from Bio-Rad Laboratories (USA) [25]. The final solution of *Pru*AA5_2A (3.5 mg/mL) was aliquoted in 50 μL fractions and submerged in liquid nitrogen for rapid freezing and stored then at -80°C.

### Activity assay, substrate range and kinetics

*Pru*AA5_2A activity was measured by following the formation of hydrogen peroxide using the previously described chromogenic ABTS (2,2'-azino-bis(3-ethylbenzothiazoline-6-sulphonic acid) and horseradish peroxidase (HRP) assay [26]. The final reaction mixture (volume: 205 μL) contained 7 U/mL horseradish peroxidase, 2 mM ABTS, and between 50 and 300 mM substrate in 20 mM MOPS buffer (pH 7.5), as the enzyme showed best performance at this pH. Prior to initiating the reaction, 5 μL of *Pru*AA5_2A (40 ng) was incubated for 30 min at 30°C in 100 μL 2x assay mix (4 mM ABTS and 15 U/mL HRP in milliQ water) to ensure complete activation of *Pru*AA5_2A by HRP. The reaction was initiated by addition of a 2x substrate concentration (in 40 mM HEPES at pH 7.5) and continuously monitored for up to 3 h by reading the absorbance at 420 nm. Hydrogen peroxide production was calculated using the extinction coefficient of the ABTS radical as described in [6].

The substrate range of *Pru*AA5_2A was determined using 300 mM D-glucose, L-arabinose, D-xylose, D-galactose, melibiose, sucrose, lactose, raffinose, stachyose, ethanol, 1-propanol, 2-propanol, 1-butanol, 1,2-butandiol, glycerol, D-sorbitol, benzyl alcohol; the exception was for the glycolaldehyde dimer, acetaldehyde, D-glyceraldehyde and glyoxalic acid where the activity was tested at 50, 25, and 15 mM of freshly prepared substrate solutions.

Kinetics parameters of *Pru*AA5_2A and *Fgr*GaOx were determined using 10 mM to 1600 mM for galactose and glycerol, 10 mM to 400 mM for raffinose and 10 mM to 150 mM for glycolaldehyde dimer using the activity assay as described above. Kinetic parameters were calculated using the Michealis-Menten function in Origin Pro 2016 (OrginLab Corp., USA), with the exception of the glycolaldehyde dimer where the substrate inhibition function was used instead of the Michaelis-Menten function.

#### Impact of pH, buffer and temperature

Effect of temperature on *Pru*AA5_2A activity was determined by performing the activity assay described above with 300 mM galactose at 25, 30, 35, 40, 50 and 60°C. The pH optimum was determined by performing the activity assay using 20 mM MOPS (pH 6.0 to 8.0), 20 mM HEPES (pH 6.0 to 8.5), or 25 mM sodium phosphate buffer (pH 6.0 to 8.0). To evaluate the impact of buffer type on the activity of *Pru*AA5_2, activity assays were also performed using following buffers and buffer concentrations: sodium phosphate (10, 25, 50 and 100 mM), potassium phosphate (10, 25 and 50 mM), HEPES (20, 40, 100 mM), MOPS (20, 40, 100 and 200 mM) and Tris-HCl buffer (20 mM) at pH 7.5.

#### Lag-phase analysis from activity data

The Gen5 microplate-reader software (BioTek, USA) was used to evaluate the lag-phase behavior of *Pru*AA5_2 as a function of buffer type and pH value, where the kinetic lag-time is time defined as the interval between the line of the inception point (maximum slope) and the absorbance baseline.

### Identification of oxidation products by MS and NMR

The negative and positive ionization MS and MS/MS spectra were produced using an Agilent 1100 Series LC/MSD Trap SL (Agilent Technologies Inc., Palo Alto, CA, USA) combined with electrospray ionization source. *Pru*AA5_2A oxidation of raffinose was performed in 200 μL sterile water with 25 mM raffinose (12.6 mg/mL), 1 U/mg raffinose of horse radish peroxide, 115 U/mg raffinose of catalase, and 1 U/mg raffinose of *Pru*AA5_2A or *Fgr*GaOx. The specific activity of *Pru*AA5_2A and *Fgr*GaOx used in these reactions was 33 U/mg and 161 U/mg, respectively. Reactions were shaken at 600 rpm for 48 hours at 30°C; samples were then diluted (1/200) in 50% methanol and 1% formic acid. To form chloride adducts in negative mode, 0.5 μL ammonium chloride was added. Samples were directly infused at 5 μL/min, and ionization parameters were as follows: drying gas 4 L/min, nebulizer pressure 10 psi, temperature 325°C and capillary voltage 3500 V.

*Pru*AA5_2A oxidation of sucrose was performed similar as described above but using 50 mM sucrose and 2 U/mg sucrose of *Pru*AA5_2A. The specific activity of *Pru*AA5_2A was 4.8 U/mg on sucrose. A sample containing the oxidation products of sucrose (2 mg) was analyzed by nuclear magnetic resonance (NMR) spectrometry. NMR measurements were performed at the ^1^H frequency of 850 MHz (sample in D_2_O) and 600 MHz (sample in DMSO) on Bruker Advance III HD spectrometers both equipped with a triple resonance cryogenic probes at 298 K.

## Results

### Comparative analysis of characterized and engineered AA5_2 sequences to inform sequence selection

**Fig 1:**
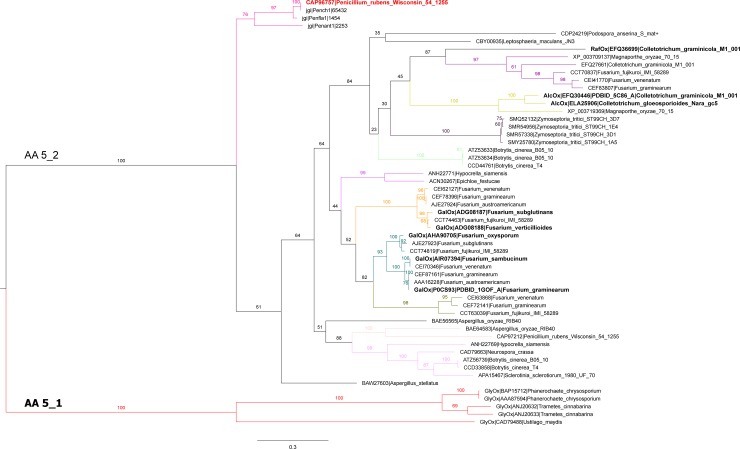
Phylogenetic tree of AA5_2. Subfamilies 1 (AA5_1) and 2 (AA5_2) are indicated. GenBank identifiers (Uniprot identifier P0CS93 in the case of the *F*. *graminearum*) are given for all sequences available in the public CAZy database [18] as of May 2018. JGI protein identifiers are given for Ascomycota homologs of *Pru*AA5_2A. Sequences for which biochemical data is available are displayed in bold and indicated as glyoxal oxidases (GlyOx) [27–31], galactose oxidases (GalOx) [26,32], general alcohol oxidases (AlcOx) [16] and raffinose oxidase (RaOx) [6]. When available the three dimensional structures are also indicated with the corresponding PDBID. Sequences were aligned using MUSCLE and the tree was constructed using RAxML v8.2.10. The robustness of the branches was assessed by the bootstrap method with 100 replications. Bootstrap values are indicated at each branch supporting the different subgroups. Subgroups were formed by exhibiting bootstrap values > 75 and colored accordingly.

A key aim of this study was to probe unexplored regions of the AA5_2 phylogeny to identify an AA5_2 member with a divergent substrate profile, which would further elucidate sequence-function relationships within this protein family. Accordingly, a phylogenetic tree was constructed which underscored the clear distinction between AA5_1 and AA5_2 members, and displayed 11 subgroups within the AA5_2 subfamily (bootstrap value >75) for which only four contain characterized members (Fig 1). *Pru*AA5_2A from *Penicillium rubens Wisconsin* is included within the AA5_2 subfamily and clusters with homologs from other *Penicillium* species in a subgroup clearly separated from the rest of the other AA5_2 sequences.

Alignment of the current AA5_2 sequences from the *Fusarium* genus, including *Fgr*GaOx, revealed 10 consensus regions in the catalytic domain of galactose oxidase (Fig A in S1 File). Corresponding consensus regions were also highly conserved within AA5_2 sequences from other organisms. For example, *Pru*AA5_2A shares nearly 50% overall sequence identity to *Fgr*GaOx, and 84% identity within consensus regions. Not surprisingly, amino acids that play a direct role in catalysis locate within the conserved sequence stretches, whereas amino acids contributing to substrate preference or catalytic efficiency mostly lie outside these regions (Table 1).

Closer inspection of the *Pru*AA5_2A primary sequence identified an N-terminal F5_F8_type_C (CBM32) domain, a central 7-bladed β-propeller (Kelch_1 repeat) catalytic domain, and a C-terminal DUF1929 domain. *Pru*AA5_2A includes the main galactose ligand arginine (Arg327), which corresponds to Arg330 in *Fgr*GaOx. However, *Pru*AA5_2A contains an aspartic acid (Asp326) at the position of Gln326 in *Fgr*GaOx, which is involved in coordinating the position of Arg330 through hydrogen bonding [33,34]. *Pru*AA5_2A also contains a serine that corresponds to the C383S substitution in *Fgr*GaOx leading to nearly five times increased catalytic efficiency [35,36]. Considering amino acid positions believed to influence substrate binding, Gln406 of *Fgr*GaOx that interacts with the C2-hydroxyl of galactose is instead glutamic acid in *Pru*AA5_2A [34,37]. *Pru*AA5_2A also contains a tyrosine in place of Phe194 in *Fgr*GaOx, which could potentially also impact substrate range [38].

**Fig 2:**
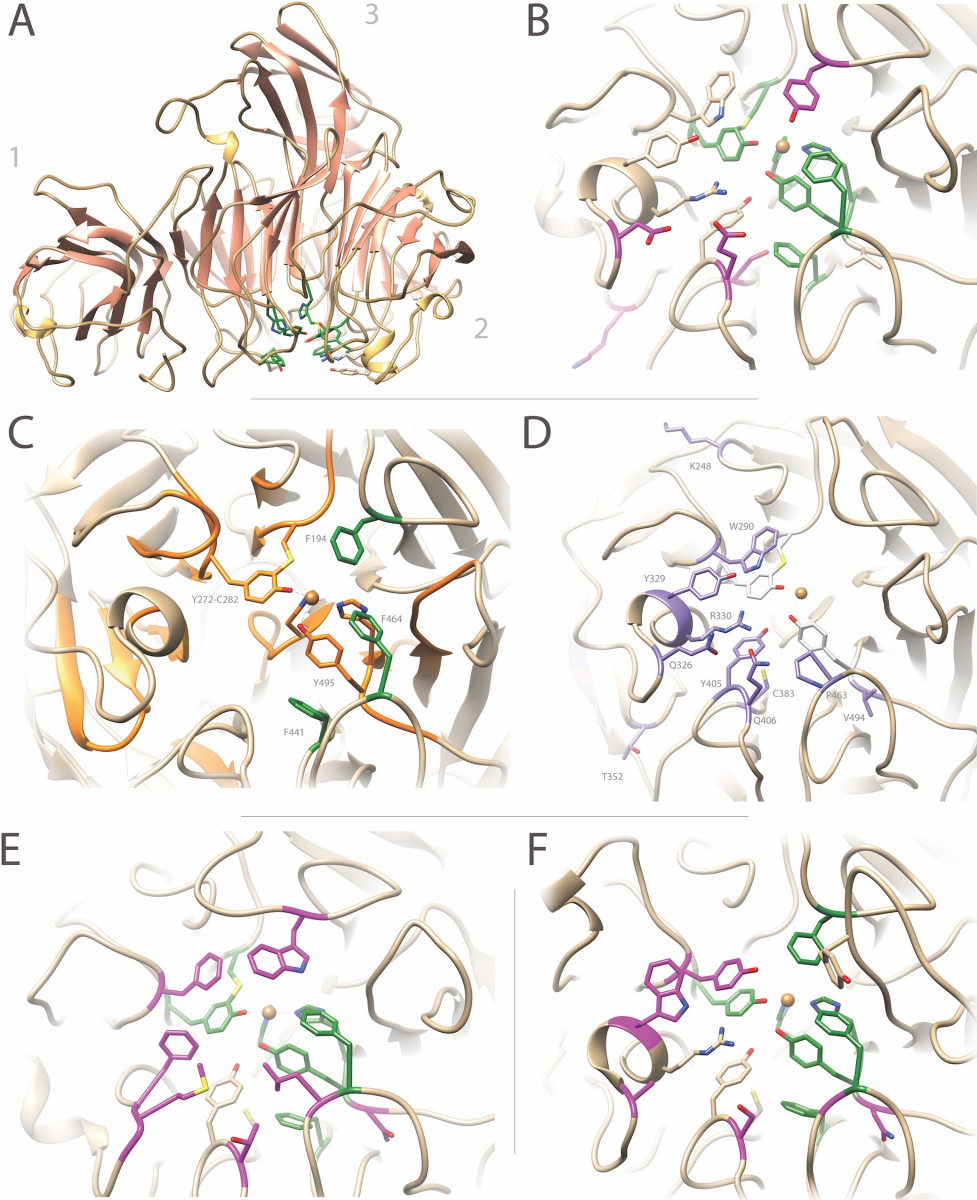
Structural comparison of *Pru*AA5_2A, *Fgr*GaOx, *Cgr*AlcOx and *Cgr*RaOx. (A) The modular structure of *Pru*AA5_2A was created using Phyre2. Similar to *Fgr*GaOx, *Pru*AA5_2A consists of (1) an N-terminal CBM32 (F5_F8_type_C) domain, (2) a central catalytic β-propeller domain and (3) a C-terminal DUF1929 domain. (B) The active site of *Pru*AA5_2A indicating conserved aromatic residues implicated in catalysis (Table 1) are shown in green; amino acids that deviate from *Fgr*GaOx are shown in magenta. (C) The active site of *Fgr*GaOx (PDBID 1GOG) containing the aromatic residues implicated in catalysis (Table 1), which are located within consensus sequence stretches around the active site (orange) (Fig A in S1 File). F194, F441 and F464 (green) do not lie in consensus sequences but are highly conserved in AA5_2. (D) Amino acids contributing to substrate preference or that affect *Fgr*GaOx performance (Table 1), are shown in purple. (E) The active site of *Cgr*AlcOx (PDB ID 5C92), which lacks all known galactose ligands in *Fgr*GaOx. Amino acids that deviate from *Fgr*GaOx are shown in magenta whereas conserved aromatic residues implicated in catalysis is shown in green. (F) Active site of the *Cgr*RaOx model. *Cgr*RaOx contains the arginine corresponding to Arg330 in *Fgr*GaOx, but lacks galactose ligands at positions corresponding to Trp290 and Qln406 in *Fgr*GaOx. Amino acids that deviate from *Fgr*GaOx are shown in magenta whereas conserved aromatic residues implicated in catalysis are shown in green.

Amino acids in *Pru*AA5_2A corresponding to those listed in Table 1 were predicted through structural modeling using the Phyre2 server; 98% of the residues were modelled at >90% confidence (Fig 2A and 2B). The stereochemical quality of the predicted model was evaluated through RAMPAGE [43], for which the Ramachandran plot predicted 98% residues lying in the most favored region. This model was compared with the crystal structures of *Fgr*GaOx (PDBID GOG1) and *Cgr*AlcOx (PDBID 5C92) and a model of the *Cgr*RaOx catalytic domain that covers 55% of the C-terminal end of the sequence with 100% confidence (Fig 2C–2F). The comparison of model and solved protein structures highlighted a region within the catalytic domain of characterized AA5_2 members that is consistently enriched in aromatic amino acids that are thought to play an important role in catalysis and stability of the copper-radical oxidase (Fig 2C; Table 1), along with the frequent substitution of residues on the opposing face that are correlated to substrate preference and catalytic performance (Fig 2D; Table 1).

The unique substitutions in *Pru*AA5_2A relative to previously characterized AA5_2 members suggested the enzyme would target galactose-containing carbohydrates but display a distinct substrate profile compared to archetypal galactose oxidases. Accordingly, *Pru*AA5_2A was selected for recombinant production and biochemical characterization.

### Production of *Pru*AA5_2A

Bioreactor cultivation and downstream purification yielded 31 mg of *Pru*AA5_2A per liter of cultivation with >90% purity (assessed by SDS-PAGE; Fig B in S1 File). Whereas the calculated molecular mass of *Pru*AA5_2A is 70 kDa, the electrophoretic molecular mass of *Pru*AA5_2A expressed in *Pichia pastoris* SMD1168H was 82 kDa, suggesting glycosylation of the protein. Indeed, 5 potential N-linked and 27 O-linked potential glycosylation sites were predicted in the *Pru*AA5_2A sequence using the GlycoEP server [44]. Recombinant AA5_2 enzymes can comprise a mixture of those lacking copper or the Tyr-Cys thioether crosslink, and the mature oxidase (Cys228-Tyr227-Cu) [45]; accordingly, the purified *Pru*AA5_2A was activated using 0.5 mM copper(II) sulfate as previously described [17,46], prior to characterization.

### General biochemical properties

**Fig 3:**
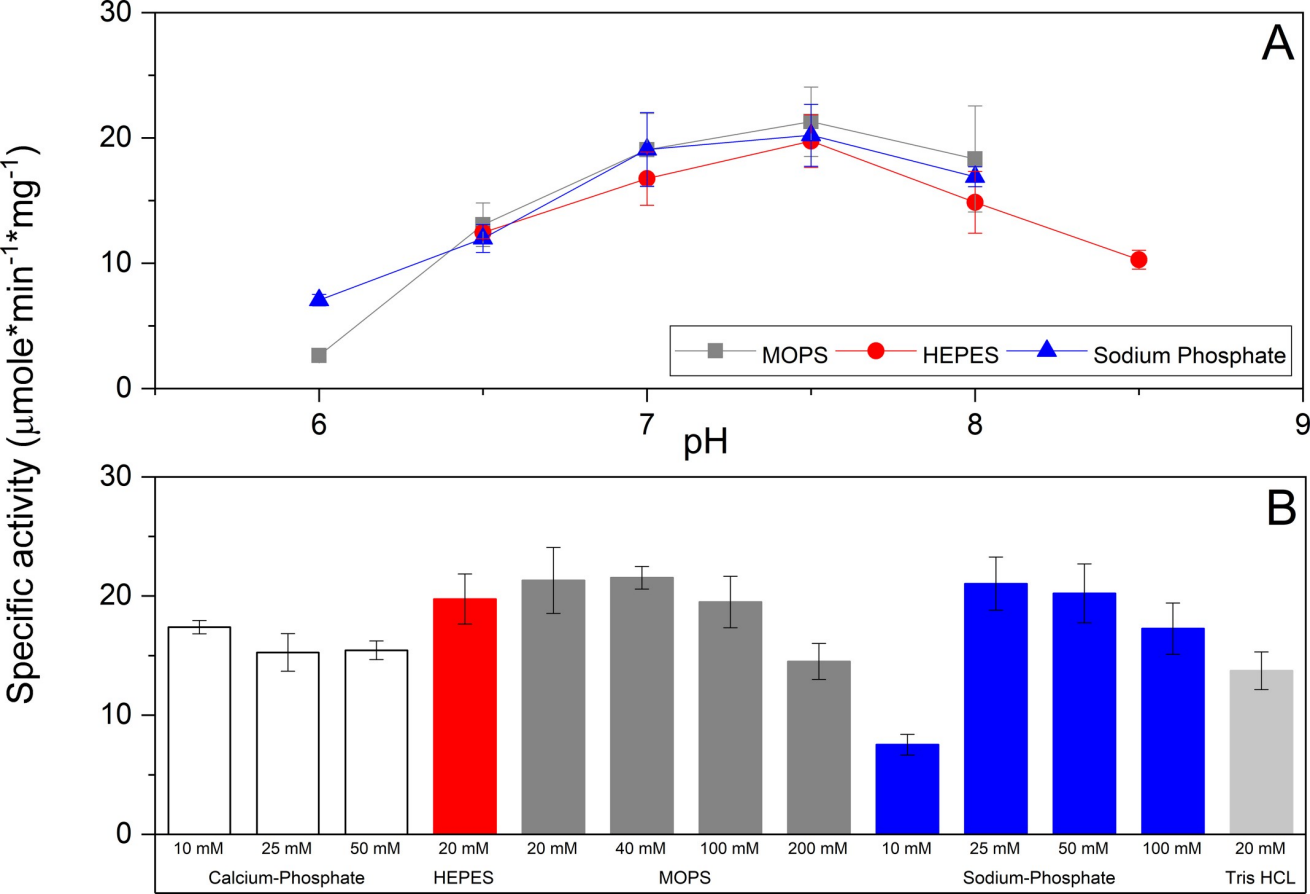
Influence of pH and choice of buffer on *Pru*AA5_2A activity. (A) Activity as a function of pH established in 25 mM MOPS, 25 mM HEPES, or 25 mM sodium phosphate. (B) Influence of buffer type and concentration on *Pru*AA5_2A activity on 150 mM raffinose (pH 7.5). n = 4; error bars indicate standard deviation.

*Pru*AA5_2A activity on galactose was optimal at pH 7.5 (Fig 3A) and the enzyme showed higher activity in HEPES or MOPS buffers compared to potassium phosphate, sodium phosphate, and Tris-Cl buffers. Specifically, *Pru*AA5_2A activity dropped by 2.7 times when increasing sodium phosphate concentration from 25 to 100 mM (Fig 3B), suggesting that phosphate ions could inhibit substrate oxidation through unfavorable interaction with the copper(II)-ion in *Pru*AA5_2A, as has been reported for other copper-containing oxidases (further discussed below). Also, *Pru*AA5_2A showed lower activity in potassium phosphate and Tris•Cl buffers relative to the sodium phosphate buffer. The highest activity of *Pru*AA5_2A was measured at 50°C; however, 70% of *Pru*AA5_2A activity was lost after eight hours at this temperature (Fig C in S1 File). Given the noted impacts of buffer type and temperature on *Pru*AA5_2A activity, all activity analyses were performed at 30°C in 20 mM MOPS (pH 7.5), unless otherwise mentioned.

**Fig 4:**
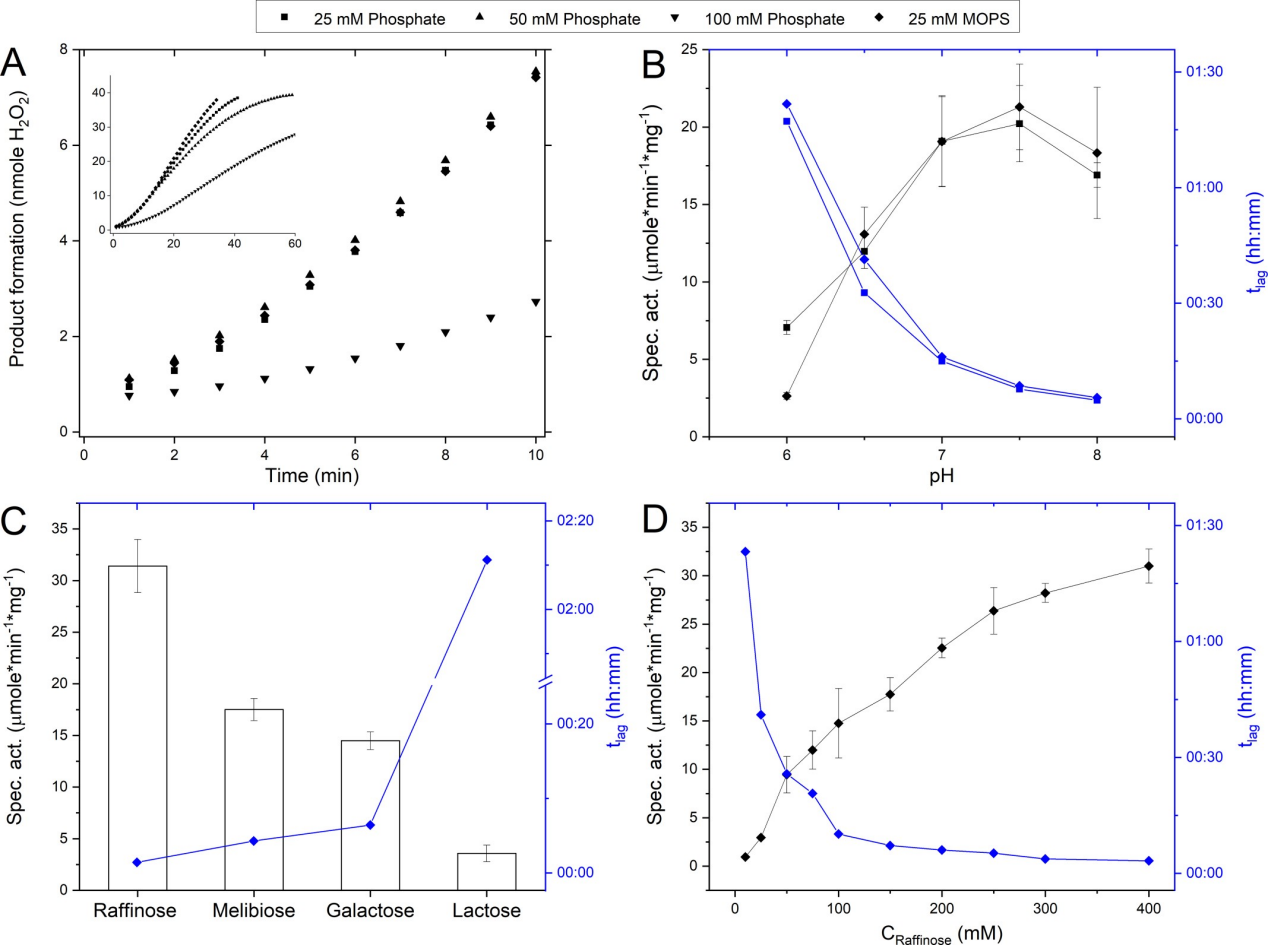
Lag-phase of *Pru*AA5_2A activity. The lag-phase (t_lag_) is defined as the time from initiation of the reaction (T = 00:00) to where the maximum slope crosses the x-axis. (A) Impact of buffer type and concentration on rate of product formation during oxidation of 300 mM raffinose (pH 7.5). (B) Impact of pH on reaction rate and t_lag_, during oxidation of 300 mM raffinose. pH was established using 25 mM sodium phosphate buffer and 25 mM MOPS. (C) Impact of substrate on reaction rate and t_lag_, where each substrate was prepared to 300 mM in 25 mM MOPS (pH 7.5). (D) Impact of substrate concentration on reaction rate and t_lag_, where raffinose was prepared in 25 mM MOPS (pH 7.5). n = 4 error bars indicate standard deviation.

For all tested conditions, *Pru*AA5_2A exhibited an initial lag-phase as shown for reactions performed in MOPS and sodium phosphate buffers (Fig 4A). In phosphate buffer (pH 7.5), the t_lag_ increased with increasing buffer concentration (Fig 4A). Treatment of *Pru*AA5_2 with 0.05 mM copper sulfate, 0.46 mM potassium ferricyanide, and 7.5 U/mL horseradish peroxidase to ensure the enzyme was in the Cys-Tyr*-Cu(II) activated state, did not diminish the t_lag_ (data not shown). Instead, t_lag_ was reduced by over 90% when shifting from pH 6.0 to pH 8.0 (Fig 4B). This impact of pH was observed for all buffers listed in Fig 3B (data not shown). Moreover, the t_lag_ was shortest for the most preferred substrates (Fig 4C) and decreased with increasing substrate concentrations (Fig 4D).

#### Substrate profile of *Pru*AA5_2A

**Fig 5:**
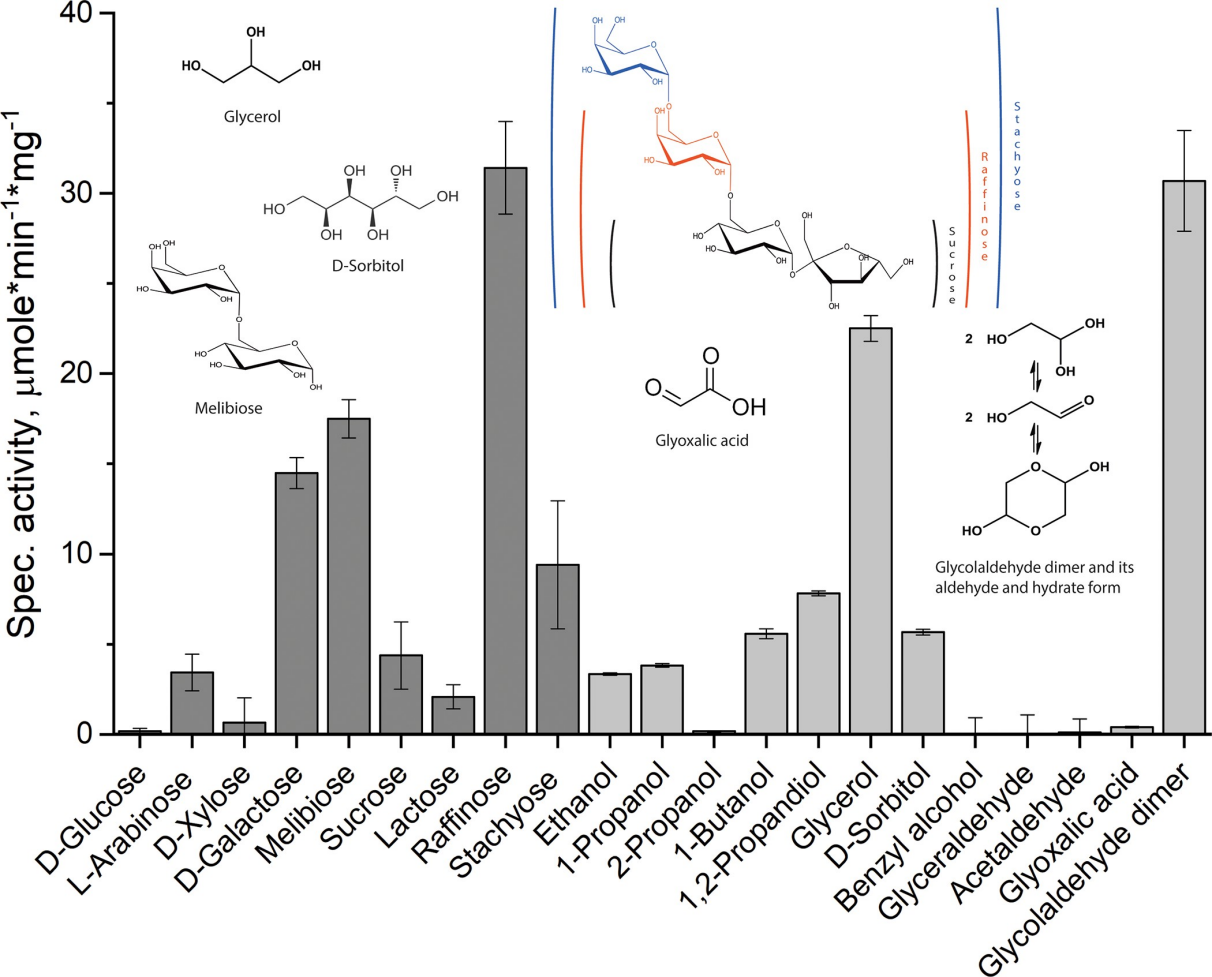
Substrate range of *Pru*AA5_2A. *Pru*AA5_2A activity was measured using 300 mM substrate, except for glyceraldehyde, acetaldehyde and glycolaldehyde dimer, where the substrate concentration was 50 mM. Activity on Glyoxalic acid was measured at 15 mM since no activity was detected at 50 mM. In all cases, reactions were performed at 30°C in 20 mM MOPS (pH 7.5). n = 4; error bars indicate standard deviation.

Of the carbohydrates tested, *Pru*AA5_2A displayed highest activity towards galactopyranosyl-α-(1–6)-substituted oligosaccharides, including raffinose (31.4 U/mg), followed by melibiose (17.5 U/mg) and then stachyose (9.4 U/mg) (Fig 5). Activity on lactose was comparatively low (2.4 U/mg), pointing to the importance of the α-(1–6)-glycoside bond of the target galactopyranosyl unit. Of note, the preference of *Pru*AA5_2A for galactopyranosyl-α-(1–6)-oligosaccharides is reminiscent of two recently discovered AA5_2 members from *Fusarium sambucinum*, *Fsa*GaOx, [47] and *Colletotrichum graminicola*, *Cgr*RaOx [6]. Moreover, despite higher activity on oligosaccharides over monosaccharides, *Pru*AA5_2A was, like *Cgr*RaOx, not active on galactose-containing polysaccharides, including galactoxyloglucan and galactoglucomannan (data not shown). Products generated by *Pru*AA5_2A oxidation of raffinose were analyzed by MS to confirm oxidation of the C-6 hydroxyl of the galactosyl residue. Identical to reaction products generated by *Fgr*GaOx and *Cgr*RaOx (see Fig D in S1 File and [6]), the main product generated by *Pru*AA5_2A was indeed oxidized at the C-6 of the galactosyl residue of raffinose (m/z 569). Like *Cgr*RaOx, the further oxidation product, carboxylic acid, was also present in minor amount (m/z 517).

Surprisingly, clear activity was found on the disaccharide sucrose (4.8 U/mg) although no activity could be detected for D-glucose. ^1^H NMR analysis was therefore conducted in both D_2_O and DMSO-d_6_ to determine whether *Pru*AA5_2A could target the fructosyl residue in sucrose [48]. Unfortunately, in both solvents, observed chemical shifts overlapped with the starting material, and thus were impossible to further analyze (Fig E in S1 File). Due to the low concentration of the product, no attempt to run 2D NMR was made.

Compounds other than carbohydrates were generally poor substrates; and similar to other AA5_2 members, *Pru*AA5_2A did not oxidize the secondary alcohol 2-propanol. The exception was for glycerol and freshly prepared solutions of glycolaldehyde dimer (50 mM), where *Pru*AA5_2A activity was 22.5 U/mg and 30.4 U/mg, respectively. Like *Cgr*RaOx, *Pru*AA5_2A activity on the glycolaldehyde dimer was only detected using freshly prepared substrate, although the specific activity of *Pru*AA5_2A was approximately 30 times higher than that of *Cgr*RaOx when compared at 50 mM substrate concentration [6]. The activity was severely diminished after overnight storage of the glycolaldehyde dimer solution and completely lost after 48 h of storage, which could be due to the gradual formation of various glycolaldehyde derivatives in solution or interference with the activity assay by glycolaldehyde (data not shown; see Fig 5 for molecular structures). Notably, *Pru*AA5_2A also exhibited a lag-phase when acting on the glycolaldehyde dimer, where t_lag_ at 50 mM of the substrate was comparable to that observed in reactions containing 300 mM raffinose (results not shown).

Other aldehydes, including D-glyceraldehyde and acetaldehyde that are also targeted by glyoxal oxidases from subfamily AA5_1, were not oxidized *Pru*AA5_2A. Low but detectable *Pru*AA5_2A activity was measured using 15 mM glyoxalic acid, which could be explained by the formation of the hydrate form (geminal diol) of the aldehyde group.

#### Kinetic properties of *Pru*AA5_2A

**Fig 6:**
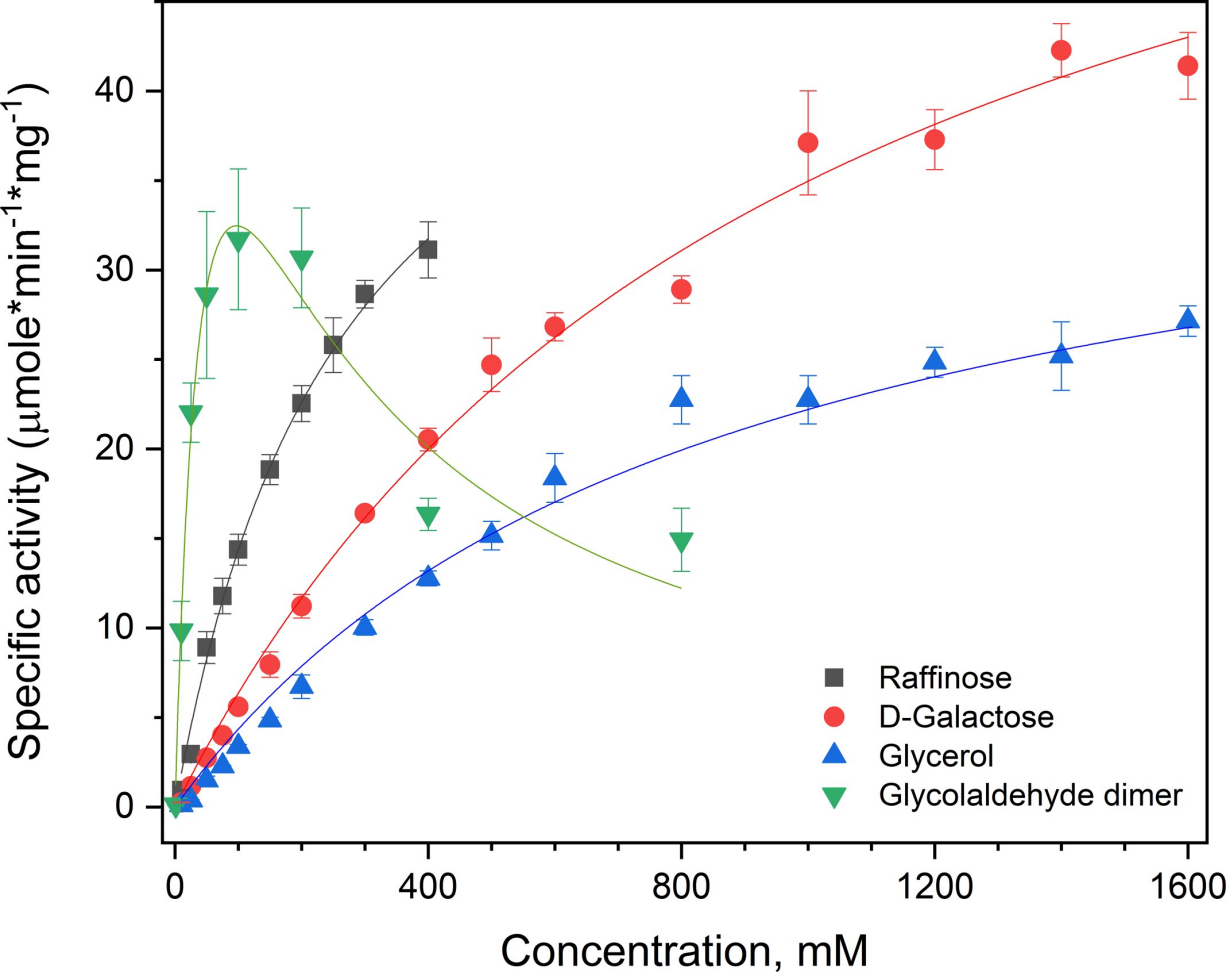
Kinetic analysis of *Pru*AA5_2A on preferred substrates. Raffinose (■), galactose (●), glycerol (▲) and a fresh solution of glycolaldehyde dimer (▼). n = 4; error bars indicate standard deviations. The data were fitted to the Michaelis-Menten or substrate-inhibition (glycolaldehyde dimer) functions using the OriginPro analysis software (iteration algorithm: Levenberberg-Marquardt); in all cases R^2^ values were > 0.95. For all substrates besides the glycolaldehyde dimer, saturation kinetics were not achieved below substrate solubility. Accordingly, apparent kinetic parameters are reported.

Given the limited range of solubility for raffinose (500 mM) and non-saturated behavior of *Pru*AA5_2A kinetic values reported herin are apparent values. Kinetic analyses using preferred substrates revealed that the apparent catalytic efficiency of *Pru*AA5_2A was nearly 2.5 times higher on raffinose compared to galactose (Fig 6). By contrast, the catalytic efficiency of *Fgr*GaOx on raffinose and galactose was similar (30 s^-1^*mM^-1^ and 20 s^-1^*mM^-1^ respectively, see Fig F in S1 File; enzyme production described in [17]). Whereas *Fgr*GaOx activity on glycolaldehyde dimer has not been detected [6], the best kinetic performance of *Pru*AA5_2A was observed using this substrate, where the apparent catalytic efficiency (*k*_cat_/*K*_M_) of *Pru*AA5_2A on freshly prepared glycolaldehyde dimer was 6.5 times higher than raffinose. The corresponding kinetic profile, however, was consistent with substrate inhibition by the glycolaldehyde dimer (*K*_i_ = 178 mM). In solution, the dimer form rearranges into glycolaldehyde and the hydrate form (Fig 5) where the hydrate form is the major component (70%) at equilibrium [49]. Given the loss of activity during storage together with the apparent substrate inhibition of glycolaldehyde dimer, it is conceivable that the hydrate form, or other derivatives of glycoladehyde, are inhibitors of *Pru*AA5_2A activity.

## Discussion

*Pru*AA5_2A represents the first family AA2_2 member retrieved from the Eurotiomycetes order to be investigated; all other characterized fungal AA5_2 members are from *Fusarium* and *Colletotrichum* species, belonging to the Sordariomycetes order. This protein was present within a distinct group in our phylogenetic study, and comparisons between *Pru*AA5_2A and other characterized fungal AA5_2 sequences highlighted amino acids that likely contribute to substrate preference (Table 1).

First, the amino acids at positions Phe194, Phe441, and Phe464 of *Fgr*GaOx are conserved among AA5_2 copper-radical oxidases and appear to play a role in stabilizing the radical electron or position of the Tyr495 copper ligand, thereby stabilizing the active form of the enzyme. These positions are in addition to Trp290, which contributes to pi-stacking interactions with the tyrocysteine linkage, but is also suggested to act as a galactose ligand [40]. In this context, it is interesting to note that residues corresponding to Trp290 and Phe194 in both *Cgr*AlcOx and *Cgl*AlcOx are swapped to phenylalanine and tryptophan, respectively (Fig 2E). By contrast, Trp290 is replaced by tyrosine and Tyr320 by tryptophan in *Cgr*RaOx (Fig 2F), whereas Phe194 is replaced by a tyrosine in *Pru*AA5_2A (Fig 2B). It is conceivable then, that a tyrosine at the edge of the active center facilitates hydrogen bonding to the glucopyranosyl unit that neighbors galactose in melibiose and raffinose, thereby increasing the selectivity of *Cgr*RaOx and *Pru*AA5_2A for these substrates. Considering the poor activity on lactose, the orientation of the glucopuranosyl also plays an important role in interaction, supporting the notion that these enzymes seem to specifically target raffinose.

Second, Arg330 and Tyr329 in *Fgr*GaOx appear critical for activity on galactose [3,34,41], and are present in *Pru*AA5_2A; however, the presence of an aspartic acid at position Gln326 (in *Fgr*GaOx) and glutamic acid at position Gln406 (in *Fgr*GaOx) could further facilitate the binding of oligosaccharides or broaden the substrate acceptance [37] (Fig 2B). Notably, positions Gln326 and Gln406 in *Fgr*GaOx are alanine and serine in *Cgr*RaOx, which might explain the comparatively poor catalytic turnover of *Cgr*RaOx on galactose and raffinose, as well as lack of detectable activity on stachyose and lactose since this enzyme seemingly lacks two of the galactose ligands (Fig 2F) [6]. Also notable, Arg330 and Tyr329 in *Fgr*GaOx are replaced by phenylalanine and methionine in *Cgr*AlcOx (Fig 2E). The new analyses of *Pru*AA5_2A, together with previous characterizations of AA5_2 oxidases, begin to suggest that the Arg330-Tyr329 pair, as well as Phe194-Trp290 pair, may delineate carbohydrate versus alcohol oxidase functionally within this enzyme subfamily.

Distinct from *Fgr*GaOx but similar to *Cgr*RaOx, *Pru*AA5_2A displayed lower *K*_M_ and higher catalytic efficiency on raffinose than galactose, and appeared unable to oxidize galactose-containing polysaccharides. Both *Pru*AA5_2A and *Cgr*RaOx are further distinguished from *Fgr*GaOx by their oxidation of the glycolaldehyde dimer. While sequence attributes leading to these activity differences were difficult to predict, it is interesting to note that the kinetic efficiency of *Pru*AA5_2A on the glycolaldehyde dimer was nearly seven times higher than tested carbohydrate substrates, where the corresponding *K*_M_ value (53 mM) is comparable to that of *Fgr*GaOx on galactose.

Lag-phases and buffer inhibition were not previously reported for other biochemically characterized AA5_2 oxidases; however, similar impacts of phosphate and Tris buffers have been observed for other metal-containing oxidoreductases. For example, inhibitory effects of phosphate and Tris buffers have been observed for iron-lipoxygenases [50,51], copper-tyrosinases [52] and copper and zinc dismutases [53]. Whereas *Pru*AA5_2A is the first wild-type AA5_2 member reported to display this phenotype, a lag phase was observed for glucose-oxidizing mutants of *Fgr*GaOx, which was ascribed to the W290F substitution and substrate-induced transformation of the W290F variant to the more active form [42]. The substrate dependence of the lag phase observed for *Pru*AA5_2A is consistent with this model, but suggests it is not only attributed to the W290F substitution, given that *Pru*AA5_2A contains a tryptophan at the corresponding position.

The biological functions of family AA5_2 oxidases remain elusive; however, conceivable options are beginning to materialize through the increasing number of AA5_2 members that display activity beyond galactose. For example, certain AA5_2 members may play a role in pathogenesis. Oxidation of glycolaldehyde may be a parthway for synthethis glyoxalic acid, which is implicated in fungal virulence [54]. Similarly, the activity of *Pru*AA5_2A and *Cgr*RaOx on raffinose points to a possible role in inhibiting oxidative stress responses in plants. Briefly, raffinose is a substrate of stachyose synthase [6,55], and stachyose along with verbascose are efficient oxygen radical scavengers in plants [56]; thus inhibition of their synthesis could weaken defense mechanisms during fungal attack. Likewise, oxidation of oligogalacturonides by AA5_2 enzymes could reduce the activiation of plant immune responses during fungal attack [57].

The comparatively high activity of *Pru*AA5_2A on the glycolaldehyde dimer solution is also reminiscent of the family AA5_1 CRO2 oxidase from *Phanerochaete chrysosporium* [58] and the copper-radical oxidase GlxA from *Streptomyces lividans* [59]. Whereas the biological role of CRO2 is unclear, family AA5_1 glyoxal oxidases (GLOXs) have already been implicated in the filamentous growth of phytopathogenic fungi [60]. GlxA does not belong to either AA5_1 or AA5_2 subclasses; however GlxA is a membrane-associated galactose-oxidase like cuproenzyme where the catalytic domain adopts a β-propeller fold and the C-terminus includes a DUF1929 domain [59]. Similar to *Pru*AA5_2A, GlxA does not accept glyoxal, shows highest activity on glycolaldehyde (*K*_M_ of 150 mM), and oxidizes galactose and glycerol albeit with *K*_M_ values above tested substrate concentrations (i.e., above 0.6 M) [59]. While the natural substrate of GlxA is unclear, GlxA contributes to β-glycan synthesis and/or modification at hypal tips [59,61], impacting aerial growth, pellet formation, and response to osmotic stress [61–63]. Orthologues of GlxA are found throughout the Streptomyces genus and are believed to have been acquired through horizontal gene transfer from fungi [62]. Accordingly, a compelling possibility is that *Pru*AA5_2A and other AA5_2 members likewise contribute to fungal cell wall remodeling.

To conclude, the dual activity of *Pru*AA5_2A on both glycolaldehyde dimer as well as carbohydrates spans the substrate range reported for AA5_1, AA5_2, and unclassified AA5 oxidases. The diversity of low molecular weight substrates accepted by *Pru*AA5_2A also reveals the potential of single AA5_2 members to contribute to multiple and diverse biological functions. The remaining difficulty in identifying the natural substrate of *Pru*AA5 and other AA5_2 members, together with the occurrence of AA5_2 sequences predomaintly in fungal plant pathogens, suggests that at least some AA5_2 members act on low-abundant molecules involved in pathogeneticty and/or defence. Localizing AA5_2 activity in corresponding fungi during pathogenesis could shead light on the biological role and preferred substrates of this enzyme family.

## Supporting information

S1 File A new family AA5_2 member displays dual activity preference. **Fig A. Analysis of AA5_2 Sequences.** (A) Sequence conservation between *Fgr*GaOx, *Pru*AA5_2A, *Cgr*RaOx and *Cgr*AlcOx in within 10 sequence stretches recognized for having identical amino acids in the 9 analyzed *Fusarium spp*. Yellow highlights identical amino acids throughout the four sequences of *Fgr*GaOx, *Pru*AA5_2A, *Cgr*RaOx and *Cgr*AlcOx. Amino acids that also occur in Table 1 are in bold, red positions denotes variances. The placements of the sequence segments in the structure of *Fgr*GaOx are highlighted in Fig 2C. (B) Sequence alignment of the 9 Fusarium spp. in subfamily AA5_2 used to identify the 10 conserved sequence segments and information presented in Table 1. The conserved sequence segments were defined as strings of 4 or more consecutive and conserved amino acids in alignment of the *Fusarium spp*. sequences.
**Fig B. SDS-PAGE of pure *Pru*AA5_2A after production and purification.** The molecular weight of *Pru*AA5_2A was estimated to be 75 kDa, indicating that the purified enzyme is glycosylated.
**Fig C. Effect of temperature on *Pru*AA5_2A activity.** (A) Activity on 300 mM raffinose in 20 mM MOPS (pH 7.5). (B) Residual activity at 30°C after 15, 30 min or 1, 2, 4 and 24 hours at 22, 30, 40, 50 and 60°C in 20 mM MOPS (pH 7.5). n = 4; error bars indicate standard deviation.
**Fig D. Analysis of oxidized products by mass spectrometry.** (A) Negative mode ESI-MS spectra of oxidized raffinose produced by *Cgr*RaOx -catalyzed reaction (3), and (B) by *Pru*AA5_2A -catalyzed reaction. m/z 517, uronic acid; m/z 539, unoxidized raffinose (Cl^-^adduct); m/z 569, aldehyde product reacted with methanol (Cl^-^adduct); m/z 207.9, MOPS buffer.
**Fig E**. ^**1**^**H NMR spectra of sucrose oxidation products.** (A) Analyzed in D_2_O, and (B) DMSO-d_6_. Chemical shifts indicating the oxidized product formation and putative oxidation sites as hydrates (A) and aldehydes (B) marked with a ring. Due to low degree of oxidation the final structure of the oxidation product could not be determined.
**Fig F. Comparative plot of *Pru*AA5_2A and *Fgr*GaOx substrate kinetics using the calculated Michealis-Menten plot from kinetical analysis.** The activity axis (y-axis) were converted to relative activity for easy comparison. Actual kinetic parameters are present in Fig 6 for *Pru*AA5_2A and the discussion for *Fgr*GaOx. No activity on glycolaldehyde dimer was detected for *Fgr*GaOx.
(PDF)
