# Supplementary material for: A family AA5_2 carbohydrate oxidase from Penicillium rubens displays functional overlap across the AA5 family
Source: PLoS One. 2019 May 15;14(5):e0216546. doi: 10.1371/journal.pone.0216546 (PMC6519835; doi:10.1371/journal.pone.0216546)
Supplement: S1 File — Fig A. Analysis of AA5_2 Sequences. (A) Sequence conservation between FgrGaOx, PruAA5_2A, CgrRaOx and CgrAlcOx in within 10 sequence stretches recognized for having identical amino acids in the 9 analyzed Fusarium spp. Yellow highlights identical amino acids throughout the four sequences of FgrGaOx, PruAA5_2A, CgrRaOx and CgrAlcOx. Amino acids that also occur in Table 1 are in bold, red positions denotes variances. The placements of the sequence segments in the structure of FgrGaOx are highlighted in Fig 2C. (B) Sequence alignment of the 9 Fusarium spp. in subfamily AA5_2 used to identify the 10 conserved sequence segments and information presented in Table 1. The conserved sequence segments were defined as strings of 4 or more consecutive and conserved amino acids in alignment of the Fusarium spp. sequences. Fig B. SDS-PAGE of pure PruAA5_2A after production and purification. The molecular weight of PruAA5_2A was estimated to be 75 kDa, indicating that the purified enzyme is glycosylated. Fig C. Effect of temperature on PruAA5_2A activity. (A) Activity on 300 mM raffinose in 20 mM MOPS (pH 7.5). (B) Residual activity at 30°C after 15, 30 min or 1, 2, 4 and 24 hours at 22, 30, 40, 50 and 60°C in 20 mM MOPS (pH 7.5). n = 4; error bars indicate standard deviation. Fig D. Analysis of oxidized products by mass spectrometry. (A) Negative mode ESI-MS spectra of oxidized raffinose produced by CgrRaOx -catalyzed reaction (3), and (B) by PruAA5_2A -catalyzed reaction. m/z 517, uronic acid; m/z 539, unoxidized raffinose (Cl-adduct); m/z 569, aldehyde product reacted with methanol (Cl-adduct); m/z 207.9, MOPS buffer. Fig E. 1H NMR spectra of sucrose oxidation products. (A) Analyzed in D2O, and (B) DMSO-d6. Chemical shifts indicating the oxidized product formation and putative oxidation sites as hydrates (A) and aldehydes (B) marked with a ring. Due to low degree of oxidation the final structure of the oxidation product could not be determined. Fig F. Comparative plot of PruAA5 [file pone.0216546.s001.pdf]

S1 File: A new family AA5_2 member displays dual activity preference

Supplemental Figures

A family AA5_2 carbohydrate oxidase from *Penicillium rubens* displays functional overlap across the AA5 family

Filip Mollerup, Ville Aumala, Kirsti Parikka, Yann Mathieu, Harry Brumer, Maija Tenkanen and Emma Master

A

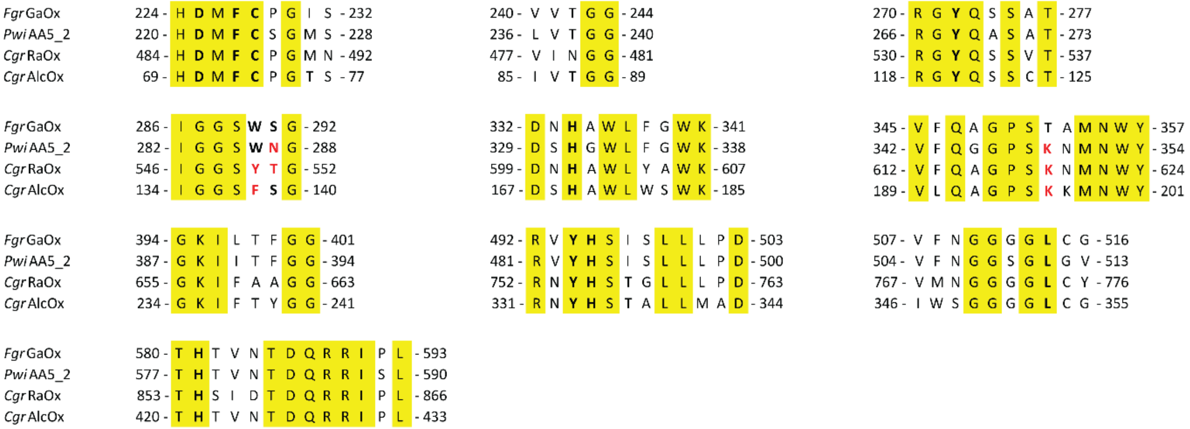

S1 File: A new family AA5_2 member displays dual activity preference

B

Alignment of CAZy family AA5_2 Fusarium spp.

```
P0CS93 GAOA_GIBZA          1   ASAPIG-SAISRNNWAVTCDSAQSGNECNKAIDGNKDTFWHTFYGAN--GDPKPPHTYTI   57
gi|167226|gb|AAA16228..    1   ASAPIG-SAISRNNWAVTCDSAQSGNECNKAIDGNKDTFWHTFYGAN--GDPKPPHTYTI   57
gi|295414058|gb|ADG08..    1   AAPPVGSNPIDRKGWTVKCSSQASNFPCGRAIDGDKNTFWQTPYGT---TNTPPPHTITI   57
gi|295414056|gb|ADG08..    1   AAPPVGSNPIDRKGWTVKCSSQAPNFPCGRAIDGDKNTFWQTPYGT---TNTPPPHTITI   57
gi|558761175|gb|AHA90..    1   ASAPIG-TAINRDKWRVTCDSQHEGDECSKAIDGDRDTFWHTAWAAGGTNDPKPPHTITI   59
gi|517324299|emb|CCT7..    1   AAPPVGSNPIDRKGWTVKCSSQASNYPCGRAIDGDKNTFWQTPYGT---TNTPPPHTITI   57
gi|517324673|emb|CCT7..    1   ASAPIG-TAISRDKWKVTCDSQHEGDECSKAIDGDRNTFWHTNWAAGGTNDPKPPHTITI   59
AIR07394.1                 1   ASAPIG-SAISRNNWAVTCDSAQPGNECNKAIDGNRDTFWHTFYGAN--GDPKPPHTYNI   57
AJE27924.1                 1   AAPPVGSNPIDRKGWTVRCSSQASNFPCGNAIDGSKDTFWQTPYGT---TNTPPPHSIVI   57
                               *: *:* . *.*. * * *.*    .  *..****.::***:* :.:    :  ***:  *

P0CS93 GAOA_GIBZA         58   DMKTTQNVNGLSMLPRQDGNQNGWIGRHEVYLSSDGTNWGSPVASGSWFADSTTKYSNFE   117
gi|167226|gb|AAA16228..   58   DMKTTQNVNGLSMLPRQDGNQNGWIGRHEVYLSSDGTNWGSPVASGSWFADSTTKYSNFE   117
gi|295414058|gb|ADG08..   58   DMKQTQYVSGLQITPRQDGNTRNWIGRHEVYLSSDGTNWGKPVAFGTYWGDKYPWITNFE   117
gi|295414056|gb|ADG08..   58   DMKQTQYVSGLQITPRQDGNTRNWIGRHEVYLSSDGTNWGKPVAFGTYWGDKYPWITNFE   117
gi|558761175|gb|AHA90..   60   DMGSSQNVNGLSVLPRQDGSDHGWIGRHNVFLSTDGKNWGDAVATGTWFADNTEKYSNFE   119
gi|517324299|emb|CCT7..   58   DMKQTQYVSGLQITPRQDGNTRNWIGRHEVYLSSDGTNWGKPVAFGTYWGDKYPWITNFE   117
gi|517324673|emb|CCT7..   60   DMGSSQNVNGLSVLPRQDGSDHGWIGRHNVFLSTDGKNWGDAVATGTWFADNTEKYSNFE   119
AIR07394.1                58   DMKTTQNVNGLSMLPRQDGSQNGWIGRHEVYLSSDGKTWGSPVATGSWFADSTTKYSNFE   117
AJE27924.1                58   DMKQTQYVSGLQITPRQDGNTRNWIGRHEVYLSSDGSNWGSPVAFGTYWGDKYPVITNFE   117
                               **  :* *.**.: *****. ..*****:*:**:**..**. ** *:::.*.    :***

P0CS93 GAOA_GIBZA        118   TRPARYVRLVAITEA-NGQPWTSIAEINVFQASSYTAPQPGLGRWGPTIDLPIVPAAAAI   176
gi|167226|gb|AAA16228..  118   TRPARYVRLVAITEA-NGQPWTSIAEINVFQASSYTAPQPGLGRWGPTIDLPIVPAAAAI   176
gi|295414058|gb|ADG08..  118   TQPARYLRFVALSNVNSDYPWIAIADFQVYNALKYNPPVKGLGKWGPTLDFPVIPVAGAV   177
gi|295414056|gb|ADG08..  118   THPARYLRFVALSNVNSDYPWIAIADFQVYNALKYNPPAKGLGKWGPTIDFPVIPVAGAV   177
gi|558761175|gb|AHA90..  120   TRPARYVRLVTVTEA-NDQPWTSIAEINVFKAASYTSPQPGLGRWGPTLDFPIVPVAAAV   178
gi|517324299|emb|CCT7..  118   TQPARYLRFVALSNVNSDYPWIAIADFVVYNALKYSPPAKGLGKWGPTLDFPVIPVAGAV   177
gi|517324673|emb|CCT7..  120   TRPARYVRLVAVTEA-NDQPWTSIAEINVFKAASYTSPQPGLGRWGPTLDFPIVPVAAAV   178
AIR07394.1               118   TRPARYVRLVALAEA-NGQPWTSIAEINVYQASSYTAPQPGLGRWGPTIDLPIVPAAAAI   176
AJE27924.1               118   TKPARYLRFVALSNVNSDNPWIAIADFVVYNALKYSPPKNGVGKWGPTLDFPVIPVAGAV   177
                               *:****:*:*::::. .. ** :**:: *::* .*. *  *:*:****:*:*::*.*.*:

P0CS93 GAOA_GIBZA        177   EPTSGRVLMWSSYRNDAFGGS--PGGITLTSSWDPSTGIVSDRTVTVTKHDMFCPGISMD   234
gi|167226|gb|AAA16228..  177   EPTSGRVLMWSSYRNDAFGGS--PGGITLTSSWDPSTGIVSDRTVTVTKHDMFCPGISMD   234
gi|295414058|gb|ADG08..  178   EPVSGKVVIWSAYRYDAFQGTTPRGGFTLTSIWDPKTNVISNRNVSNNHHDMFCPGISMD   237
gi|295414056|gb|ADG08..  178   EPVSGKVVIWSAYRYDAFQGTTPRGGFTLTSIWDPKTNVISNRNVSNNHHDMFCPGISMD   237
gi|558761175|gb|AHA90..  179   EPTSGKVLVWSSYRNDAFGGS--PGGVTLTSTWDPSTGVISQRTVTVTKHDMFCPGISMD   236
gi|517324299|emb|CCT7..  178   EPVSGKVVIWSAYRYDAFQGTTPRGGFTLTSIWDPKTNVISNRNVSNNHHDMFCPGISMD   237
gi|517324673|emb|CCT7..  179   EPTSGKVLVWSSYRNDAFGGS--PGGVTLTSTWDPSTGVISQRTVTVTKHDMFCPGISMD   236
AIR07394.1               177   EPTSGRVLMWSSYRNDAFGGS--PGGVTLTSSWDPSSGIVSDRTVTVTKHDMFCPGISMD   234
AJE27924.1               178   EPVSGKVVIWSAYRYDAFQGTNPRGGFTLTSIWDPKTNVISNRNVTNNKHDMFCPGISMD   237
                               **.**:*::**:** *** *:   **.**** ***.:.::*:*.*: .:***********

P0CS93 GAOA_GIBZA        235   GNGQIVVTGGNDAKKTSLYDSSSDSWIPGPDMQVARGYQSSATMSDGRVFTIGGSWSGGV   294
gi|167226|gb|AAA16228..  235   GNGQIVVTGGNDAKKTSLYDSSSDSWIPGPDMQVARGYQSSATMSDGRVFTIGGSWSGGV   294
gi|295414058|gb|ADG08..  238   GEGQIVVTGGNDAKKTTILMP-DGNWVPGPDMQIARGYQSSATCSDGRVFTIGGSWSGPR   296
gi|295414056|gb|ADG08..  238   GEGQIVVTGGNDAKKTTILMP-DGNWVPGPDMQIARGYQSSATCSDGRVFTIGGSWSGAR   296
gi|558761175|gb|AHA90..  237   GNGQVVVTGGNDAQKTSLYDSSSDSWIPGPDMKVARGYQSSATLSNGRVFTIGGSWSGGI   296
gi|517324299|emb|CCT7..  238   GEGQIVVTGGNDAKKTTILMP-DGNWVPGPDMQIARGYQSSATCSDGRVFTIGGSWSGPR   296
gi|517324673|emb|CCT7..  237   GSGQVVVTGGNNAEKTSLYDSSSDSWIPGPDMKVARGYQSSATLSNGRVFTIGGSWSGGI   296
AIR07394.1               235   GNGQIVVTGGNDAKKTSLYDSPSDSWVPGPDMQVARGYQSSATMSDGRVFTIGGSWSGGI   294
AJE27924.1               238   GEGQIVVTGGNDAKKTTILNP-NGEWVPGPDMQIARGYQSSATTSDGRVFTIGGSWSGPR   296
                               *.**:******:*:**::    ...*:*****::********* *:***********

P0CS93 GAOA_GIBZA        295   FEKNGEVYSPSSKTWTSLPNAKVNPMLTADKQGLYRSDNHAWLFGWKKGSVFQAGPSTAM   354
gi|167226|gb|AAA16228..  295   FEKNGEVYSPSSKTWTSLPNAKVNPMLTADKQGLYRSDNHAWLFGWKKGSVFQAGPSTAM   354
gi|295414058|gb|ADG08..  297   GGKNGEIYDPKAKTWTSLPKCLVGPMLTKDKEGVYKSDNHAWLFGWKKNSVFQAGPSTAM   356
gi|295414056|gb|ADG08..  297   GGKNGEIYDPRAKTWTSLPKCLVGPMLTHDKEGVYKADNHAWLFGWKKGSVFQAGPSTAM   356
gi|558761175|gb|AHA90..  297   FEKNGEVYDPSSKTWTSLPGALVKPMLTADQQGLYRSDNHGWLFGWKKGSVFQAGPSTAM   356
gi|517324299|emb|CCT7..  297   GGKNGEIYDPKAKTWTSLPKCLVGPMLTKDKEGVYKADNHAWLFGWKKNSVFQAGPSTAM   356
gi|517324673|emb|CCT7..  297   FEKNGEVYDPSSKTWTSLPGALVKPMLTADQQGLYRSDNHGWLFGWKEGSVFQAGPSTAM   356
AIR07394.1               295   FEKNGEVYSPSSKTWTSLPNAKVNPMLTADKQGLYRSDNHAWLFGWKKGSVFQAGPSTAM   354
AJE27924.1               297   GGKNGEIYDPKARTWTSLPKCLVGPMLTKDKEGVYKADNHAWLFGWKKGSVFQAGPSTAM   356
                                 ****:*.* ::****** . * **** *::*:*::***.******:.***********

P0CS93 GAOA_GIBZA        355   NWYYTSG--SGDVKSAGKRQSNRGVAPDAMCGNAVMYDAVKGKILTFGGSPDYQDSDATT   412
gi|167226|gb|AAA16228..  355   NWYYTSG--SGDVKSAGKRQSNRGVAPDAMCGNAVMYDAVKGKILTFGGSPDYQDSDATT   412
gi|295414058|gb|ADG08..  357   NWYYTTKGTQGDTKAAGTRRKNGRIDPDSMNGNVAMFDALKGKILTFGGATSYQQAPATA   416
gi|295414056|gb|ADG08..  357   NWYYTDRGTQGNTKAAGTRRKNGRVDPDSMNGNVAMFDAVKGKILTFGGATSYQQAPATA   416
gi|558761175|gb|AHA90..  357   NWYYTTG--NGDVKSAGKRQSSRGTDPDAMCGNAVMYDAVKGKILTFGGSPSYQDSDATT   414
gi|517324299|emb|CCT7..  357   NWYYTTRGTQGDTKAAGTRRKNGRVDPDSMNGNVAMFDALKGKILTFGGATSYQQAPATA   416
gi|517324673|emb|CCT7..  357   NWYYTSG--SGATKSAGKRQSSRGTDPDAMCGNAVMYDAVKGKILTFGGSPSYQDSDATT   414
AIR07394.1               355   NWYYTSG--SGDVKSAGKRQSNRGVAPDAMCGNAVMYDAVQGKILTFGGSPDYQDSDATT   412
AJE27924.1               357   NWYYTTRGTQGDTKAAGTRRKNGRVDPDSMNGNCVMYDALDGKILTYGGATSYQQAPATA   416
                               *****    .* .*:**.*:..    **:* ** .*:**:.*****:**: .**:: **:

P0CS93 GAOA_GIBZA        413   NAHIITLGEPGTSPNTVFASN--GLYFARTFHTSVVLPDGSTFITGGQRRGIPFEDSTPV   470
gi|167226|gb|AAA16228..  413   NAHIITLGEPGTSPNTVFASN--GLYFARTFHTSVVLPDGSTFITGGQRRGIPFEDSTPV   470
gi|295414058|gb|ADG08..  417   NAHVLTIDQPGAIAQTALVGNNGAGIHARVFATSVILPDGNVFITGGQSYSNPFTDTNAQ   476
gi|295414056|gb|ADG08..  417   NAHVLTIDQPGAIAQTALVGNNGAGIHARVFATSTILPDGNVFITGGQSYSNPFTDTNAQ   476
gi|558761175|gb|AHA90..  415   NAHIITISEPGSTPKTVFASN--GLYYPRTFHTSVILPDGNVFITGGQQRGIPFADSTPQ   472
gi|517324299|emb|CCT7..  417   NAHVLTIDEPGAIAQTALVGNNGAGIHARVFATSVILPDGNVFITGGQSYSNPFTDTNAQ   476
gi|517324673|emb|CCT7..  415   NAHIITIGEPGSTPKTVFASN--GLYYPRTFHTSVILPDGNVFITGGQQRGIPFADSTPQ   472
AIR07394.1               413   NAHIITLGEPGSTPKTVFASN--GLYFARTFHTSVVLPDGSTFITGGQRRGIPFEDSTPV   470
AJE27924.1               417   NAHVLAIAEPGAIAQTYLVGNNGAGNYARVFHTSVVLPDGNVFITGGQSYSNPFTDTNAQ   476
                               ***:::: :**:  :* :..*  .  . *.* **.:****..******  . ** *:.

P0CS93 GAOA_GIBZA        471   FTPEIYVPEQDTFYKQNPNSIVRVYHSISLLLPDGRVFNGGGGLCGDCTTNHFDAQIFTP   530
gi|167226|gb|AAA16228..  471   FTPEIYVPEQDTFYKQNPNSIVRVYHSISLLLPDGRVFNGGGGLCGDCTTNHFDAQIFTP   530
gi|295414058|gb|ADG08..  477   LEPEMFISSSNTFAKQQSNTIPRTYHSMSLLLPDATVFNGGGGLCGSCKSNHFDAQIFTP   536
gi|295414056|gb|ADG08..  477   LEPEMFISSSNTFTKQQPNTIPRTYHSMSLLLPDGTVFNGGGGLCGSCKSNHFDAQIFTP   536
gi|558761175|gb|AHA90..  473   LTPELYVPNDDTFYKQQPNSIVRVYHSISLLLPDGRVFNGGGGLCGDCDTNHFDAQIYTP   532
gi|517324299|emb|CCT7..  477   LEPEMFVSSSNTFTKQQPNTIPRTYHSMSLLLPDATVFNGGGGLCGSCKSNHFDAQIFTP   536
gi|517324673|emb|CCT7..  473   LTPELYVPNDDTFYKQQPNSIVRVYHSISLLLPDGRVFNGGGGLCGDCDTNHFDAQIYTP   532
AIR07394.1               471   LTPEIYVPEQDTFYKQNPNSIVRVYHSISLLLPDGRVFNGGGGLCGDCTTNHFDAQIFTP   530
AJE27924.1               477   LTPEMYIPTTHEFKTQQPNTIPRTYHSMSLLLPDATVFNGGGGLCGSCSSNHFDAQIYTP   536
                               : **:::   . * .*: *:* *.***:******. **********.* :*******:**

P0CS93 GAOA_GIBZA        531   NYLYNSNGNLATRPKITRTSTQSVKVGGRITISTDSSISKASLIRYGTATHTVNTDQRRI   590
gi|167226|gb|AAA16228..  531   NYLYNSNGNLATRPKITRTSTQSVKVGGRITISTDSSISKASLIRYGTATHTVNTDQRRI   590
gi|295414058|gb|ADG08..  537   QYLLDGNGNLATRPKITAVSATTAKVGSTITVTANSAIKSASLIRYGTATHVVNTDQRRI   596
gi|295414056|gb|ADG08..  537   QYLLDGNGNLATRPKITAVSATTAKVGSTITVTANSAIKSASLMRYGTATHVVNTDQRRI   596
gi|558761175|gb|AHA90..  533   NNLYDSNGKLATRPKITKVSAKSVKVGGKITITADTSIKQASLIRYGTSTHTVNTDQRRI   592
gi|517324299|emb|CCT7..  537   QYLLDGNGNLATRPKITAVSATTAKVGSTITVTANSAIKSASLIRYGTATHVVNTDQRRI   596
gi|517324673|emb|CCT7..  533   NNLYDSSGKLATRPKITKVSAKSVKVGGKITISTNTSIKQASLIRYGTSTHTVNTDQRRI   592
AIR07394.1               531   NYLYDSNGNLATRPKITRTSTQSVKVGGRVTISTDYSIRKASLIRYGTATHTVNTDQRRI   590
AJE27924.1               537   QYLLDGNGNLATRPKITAVSATTAKIGSTITVTANSAIKSASLIRYGTATHTVNTDQRRI   596
                               : * :..*:******** .*: :.*:*. :*:::: :* .***:****:**.********

P0CS93 GAOA_GIBZA        591   PLTLTNN-GGNSYSFQVPSDSGVALPGYWMLFVMNSAGVPSVASTIRVTQ   639
gi|167226|gb|AAA16228..  591   PLTLTNN-GGNSYSFQVPSDSGVALPGYWMLFVMNSAGVPSVASTIRVTQ   639
gi|295414058|gb|ADG08..  597   PLALTGA-GTNKYSFKIPNDSGIALPGYWMLFVLNNAGVPSVASTIKVTV   645
gi|295414056|gb|ADG08..  597   PLALTGA-GTNKYSFKIPNDSGIALPGYWMLFVINNAGVPSVASTIKVTV   645
gi|558761175|gb|AHA90..  593   PLSLRRTGTGNSYSFQVPSDSGIALPGYWMLFVMNSAGVPSVASTLLVTQ   642
gi|517324299|emb|CCT7..  597   PLALTGA-GTNKYSFKIPNDSGIALPGYWMLFVLNNAGVPSVASTIKVTN   645
gi|517324673|emb|CCT7..  593   PLSLRSTGSGNSYSFQVPSDSGIALPGYWMLFVINSAGVPSVASTLLVTQ   642
AIR07394.1               591   PLTLMNN-GGNSYSFQVPSDTGIALPGYWMLFVINSAGVPSVASTIRVTQ   639
AJE27924.1               597   PLALTGA-GTNKYSFKIPNDSGIALPGYWMLFVLNNAGVPSVASTIKVTV   645
                               **:*     *.***::*.*:*:**********:*.*********: **
```

S1 File: A new family AA5_2 member displays dual activity preference

Fig A. Analysis of AA5_2 Sequences. (A) Sequence conservation between *Fgr*GaOx, *Pru*AA5_2A, *Cgr*RaOx and *Cgr*AlcOx in within 10 sequence stretches recognized for having identical amino acids in the 9 analyzed *Fusarium spp*. Yellow highlights identical amino acids throughout the four sequences of *Fgr*GaOx, *Pru*AA5_2A, *Cgr*RaOx and *Cgr*AlcOx. Amino acids that also occur in Table 1 are in bold, red positions denotes variances. The placements of the sequence segments in the structure of *Fgr*GaOx are highlighted in Figure 2C. (B) Sequence alignment of the 9 Fusarium spp. in subfamily AA5_2 used to identify the 10 conserved sequence segments and information presented in Table 1. The conserved sequence segments were defined as strings of 4 or more consecutive and conserved amino acids in alignment of the *Fusarium spp.* sequences.

S1 File: A new family AA5_2 member displays dual activity preference

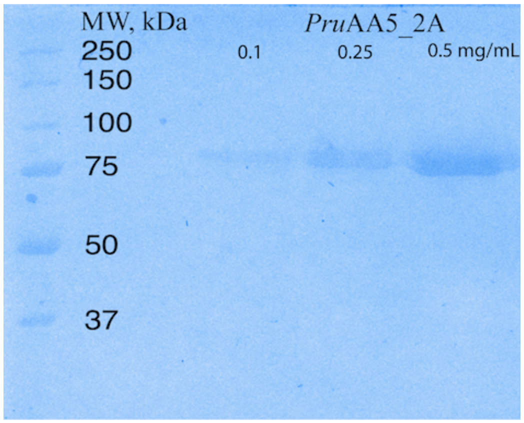

Fig B. SDS-PAGE of pure *Pru*AA5_2A after production and purification. The molecular weight of *Pru*AA5_2A was estimated to be 75 kDa, indicating that the purified enzyme is glycosylated.

S1 File: A new family AA5_2 member displays dual activity preference

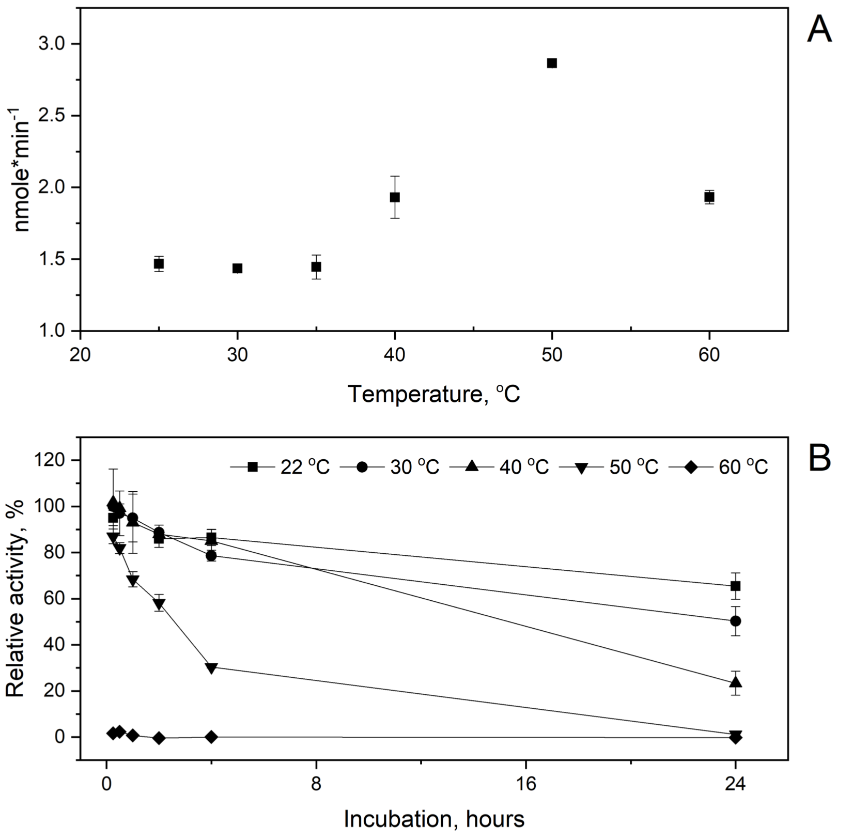

Fig C. Effect of temperature on *Pru*AA5_2A activity. (A) Activity on 300 mM raffinose in 20 mM MOPS (pH 7.5). (B) Residual activity at 30 °C after 15, 30 min or 1, 2, 4 and 24 hours at 22, 30, 40, 50 and 60 °C in 20 mM MOPS (pH 7.5). n=4; error bars indicate standard deviation.

S1 File: A new family AA5_2 member displays dual activity preference

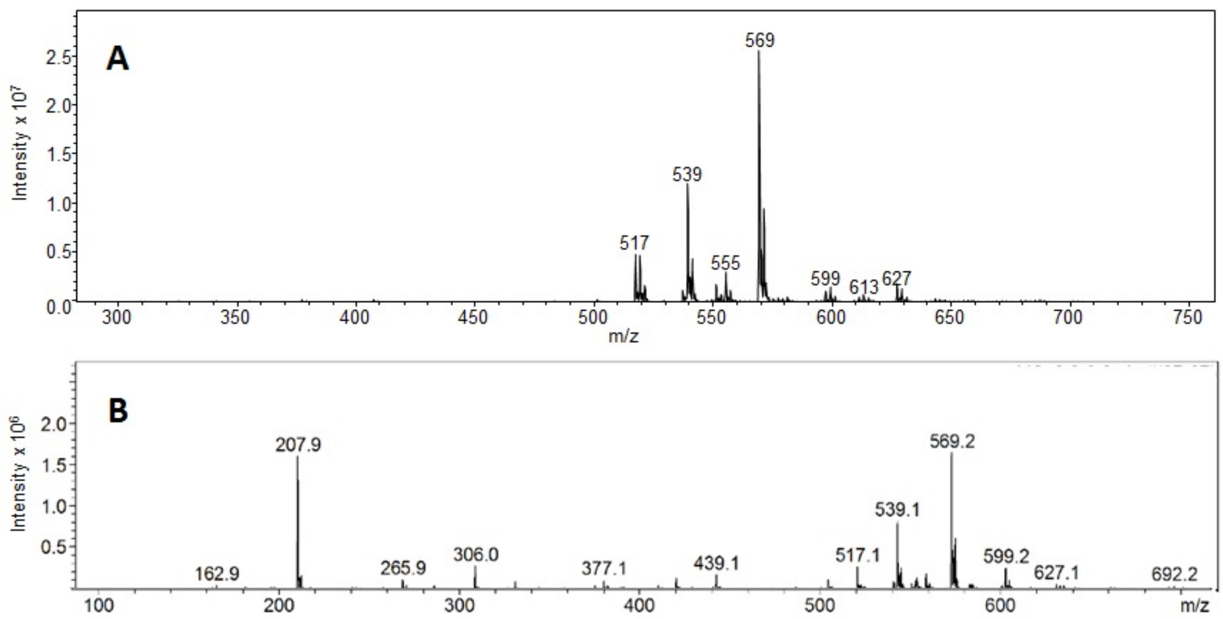

Fig D. Analysis of oxidized products by mass spectrometry. (A) Negative mode ESI-MS spectra of oxidized raffinose produced by *Cgr*RaOx -catalyzed reaction (3), and (B) by *Pru*AA5_2A -catalyzed reaction. m/z 517, uronic acid; m/z 539, unoxidized raffinose (Cl^- adduct); m/z 569, aldehyde product reacted with methanol (Cl^-adduct); m/z 207.9, MOPS buffer.

S1 File: A new family AA5_2 member displays dual activity preference

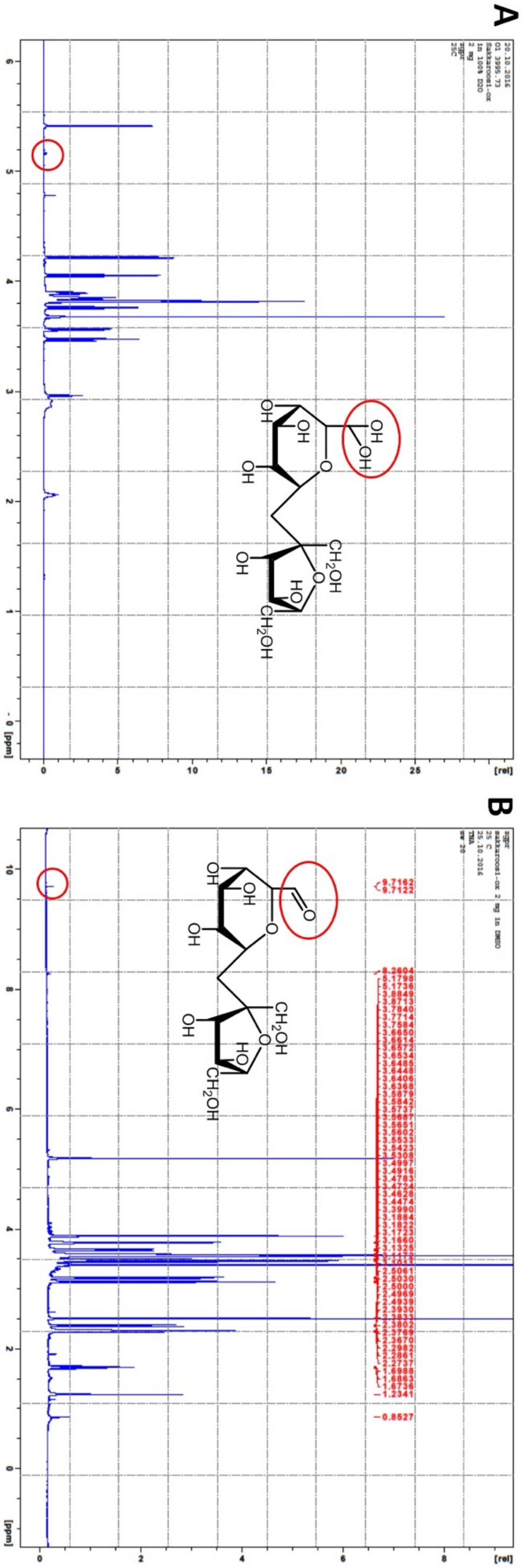

S1 File: A new family AA5_2 member displays dual activity preference

Fig E. ^{1}H NMR spectra of sucrose oxidation products. (A) Analyzed in D_2O, and (B) DMSO-d_6. Chemical shifts indicating the oxidized product formation and putative oxidation sites as hydrates (A) and aldehydes (B) marked with a ring. Due to low degree of oxidation the final structure of the oxidation product could not be determined.

S1 File: A new family AA5_2 member displays dual activity preference

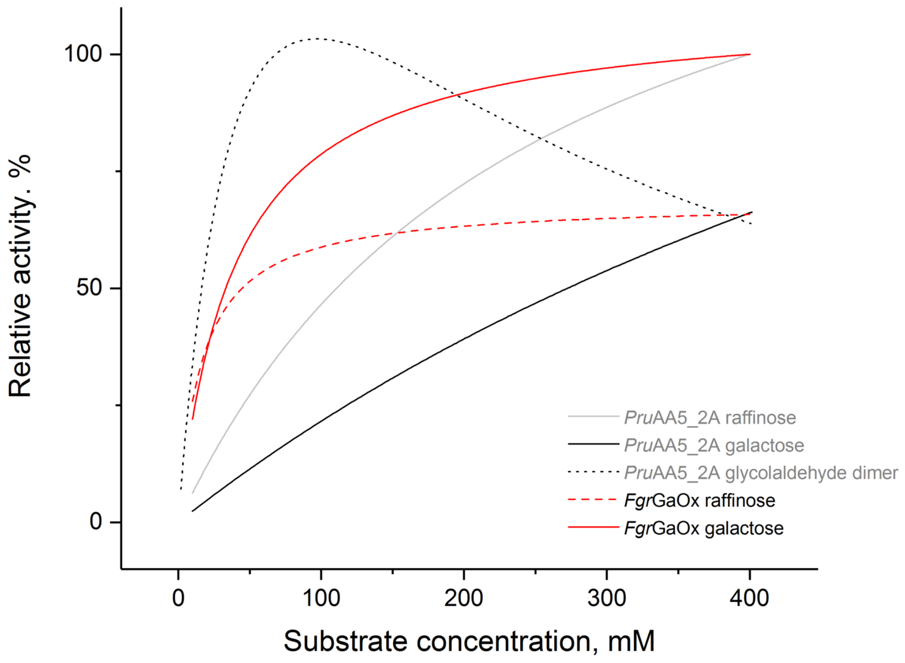

Fig F. Comparative plot of *Pru*AA5_2A and *Fgr*GaOx substrate kinetics using the calculated Michealis-Menten plot from kinetical analysis. The activity axis (y-axis) were converted to relative activity for easy comparison. Actual kinetic parameters are present in Fig 6 for *Pru*AA5_2A and the discussion for *Fgr*GaOx. No activity on glycolaldehyde dimer was detected for *Fgr*GaOx.
